# p53 Represses Transcription of RING Finger LIM Domain-Binding Protein RLIM through Sp1

**DOI:** 10.1371/journal.pone.0062832

**Published:** 2013-05-01

**Authors:** Xiangtao Kong, Bo Peng, Yang Yang, Pingzhao Zhang, Bo Qin, Dingding Han, Chenji Wang, Yongjun Dang, Jun O. Liu, Long Yu

**Affiliations:** 1 State Key Laboratory of Genetic Engineering, Institute of Genetics, School of Life Sciences, Fudan University, Shanghai, P.R. China; 2 Institutes of Biomedical Sciences, Fudan University, Shanghai, P. R. China; 3 Departments of Pharmacology and Oncology, The Johns Hopkins School of Medicine, Baltimore, Maryland, United States of America; 4 Division of Oncology Research, Department of Oncology, Mayo Clinic, Rochester, Minnesota, United States of America; Cardiff University, United Kingdom

## Abstract

RLIM acts as a negative regulator of LIM-Homeodomain proteins either by recruiting Sin3A/Histone Deacetylase (HDAC) co-repressor complex or through degradation of CLIM coactivator, thus playing an important role in embryonic development. Recent studies by different research groups have shown that RLIM acts as an X-encoded, dose-dependent inducer of X chromosome inactivation in mouse embryonic stem cells. However, until now, very little is known about the expression regulation of *RLIM* gene, and we tried to study the transctriptional regulation of *RLIM* gene. In the present study, we identified RLIM as a novel target of p53 and demonstrated that p53 repressed both mRNA and protein levels of RLIM. Expression of wild type p53, but not p53 mutants, led to repression of the RLIM promoter activity. We further identified four putative Sp1 elements (S1 to S4) on the RLIM promoter that are essential for p53-mediated repression of RLIM. Although p53 does not directly bind to the RLIM promoter, it physically interacts with and prevents the binding of Sp1 to the RLIM promoter. Thus, RLIM is a novel target of p53, and p53 exerts its inhibitory effect on RLIM expression by interfering with Sp1-mediated transcriptional activation on RLIM. Our results provided data to enlarge the knowledge of transcriptional regulation of RLIM and suggested a new pathway by which physiological and pathological activators of p53 may affect development.

## Introduction

The p53 tumor suppressor is known as the “guardian of the genome” because of its critical role in tumor suppression [1]. Around 50% of human cancers carry mutated p53, and many human tumors with wild type p53 are often defective in either activating or responding to p53 [2]. The consensus p53 DNA-binding sequence (RE) consists of two repeats of the 10-bp head-to-head arranged motif 5′-PuPuPuC(A/T)- (T/A)GPyPyPy-3′ separated by 0–13 nucleotides [3]. As a sequence-specific DNA-binding protein, p53 functions by either activating or repressing the expression of target genes. The expression of p53 is under tight regulation. In normal cells, p53 is expressed at low levels. In response to various types of stress, the steady-state levels and transcriptional activity of p53 increase dramatically, leading to the transcriptional regulation of the target genes which in turn induce cell cycle arrest or apoptosis [4].

Although p53 is a well-established transcription activator, emerging evidence suggests that p53 is also capable of repressing the transcription of target genes. The mechanisms of p53-mediated transrepression include interference with the functions of transcriptional activators (such as Sp1, ETS1) or the basal transcriptional machinery, recruitment of the histone deacetylases, chromatin remodeling, and binding of p53 to a novel type of “repression site” RE [5].

The LIM domain functions as a modular domain to mediate protein-protein interactions. LIM domain proteins can be classified into four broad categories including LIM-Homeodomain (LIM-HD) proteins, LIM only (LMO) proteins, LIM actin associated proteins and LIM catalytic proteins [6]. The LIM-HD proteins constitute a superfamily of transcription factors that interact with other transcriptional regulators in a homodimeric or heterodimeric fashion through LIM domains. And they act in a myriad of biological progresses such as development of the nervous system, cell-fate determination and tissue-specific gene expression [7], [8].

LIM-HD family of transcription factors is subject to regulation by both coactivators and corepressors. CLIM/LDB is the coactivator of LIM-HD proteins, which can overcome the inhibitory actions of the LIM domain on LIM-HD proteins and are required for LIM-HD proteins to exert their transcriptional and biological activity [9]. The intrinsic dimerization capacity of CLIM allows LIM-HD proteins to interact with distinct transcriptional regulatory proteins, thus increasing transcriptional activity of LIM-HD proteins [10].

The RING finger LIM domain-binding protein (RLIM) encoded by the *RLIM* gene acts as a negative coregulator for LIM-HD transcription factors LHX2, LHX3 and LMO2 via the recruitment of the Sin3A/histone deacetylase (HDAC) corepressor complex [11]. LHX2 has been shown to regulate chick limb development and the repression of LHX2 by RLIM contributes to the control of embryonic development [11]. In addition to recruiting Sin3/HDACs to LIM-HD, RLIM has also been shown to act as an E3 ubiquitin ligase, targeting CLIM for degradation through the RING domain of RLIM [12]. Thus, RLIM exerts inhibitory effects on LIM-HD by two distinct and complementary mechanisms - recruitment of Sin3A/HDAC or degradation of CLIM coactivator. Recently, studies by different research groups have shown that RLIM acts as an X-encoded, dose-dependent inducer of X chromosome inactivation (XCI) in mouse embryonic stem cells [13], [14]. The above data suggest broad and important role of RLIM.

Putative binding sites for several transcription factors have been identified in the proximal promoter region of mouse *RLIM* gene which encodes RLIM protein; they include C/EBPα, Sp1, Sox and RBP-J [15]. There is significant conservation between human, mouse and chicken *RLIM* gene promoters [15]. However, until now, very little is known about the transcriptional regulation of *RLIM* gene.

In the present study, we identified p53 as a negative regulator of RLIM. Wild type p53 can repress the luciferase reporter under the control of the −500/+100 region of RLIM promoter, whereas p53 mutants could not. Induction of endogenous p53 by DNA damaging agents repressed both mRNA and protein levels of endogenous RLIM. Knockdown of endogenous p53 by siRNA led to increase of endogenous RLIM level. Furthermore, the inhibitory effect of p53 on RLIM was found to be mediated by transcriptional activator Sp1. Deletions or mutations of Sp1 elements on the RLIM promoter profoundly abrogated the repression of RLIM by p53. ChIP and EMSA experiments demonstrated the direct binding of Sp1 transcription factor to the RLIM promoter both *in vivo* and *in vitro*, and the addition of p53 protein significantly inhibited the binding of Sp1 to the RLIM promoter, which explained the repression of RLIM by p53.

## Materials and Methods

### Cell Lines

The human lung carcinoma cell line H1299 (p53^−/−^) and human hepatocellular carcinoma cell line Hep3B (p53^−/−^) were cultured in RPMI 1640 medium supplemented with 10% fetal bovine serum. The human osteogenic sarcoma cell line U2-OS (p53^+/+^) was cultured in McCoy’s 5A medium supplemented with 10% fetal bovine serum. The cell line Hela (p53^+/+^) was cultured in Dulbecco’s modified Eagle’s medium with 10% fetal bovine serum. All the cell lines were obtained from the American Type Culture Collection (ATCC) and cultured in 5% CO_2_ at 37°C.

### Plasmid Constructs

The pCMV-Myc-Sp1 and pCMV-Myc-RLIM plasmids were generated by inserting the CDS of Sp1 or RLIM into the pCMV-Myc vector (Clontech). The p53 cDNA was cloned into the pCMV-HA vector (Clontech) and pGEX-4T-1 vector (Amersham) to generate the pCMV-HA-p53 and pGEX-4T-1-p53 plasmids, respectively. The p53 mutant constructs pCMV-HA-p53-R175H, R248W, R273H and R282W were generated by site-directed mutagenesis with the QuikChange Site-Directed Mutagenesis Kit (Stratagene). All the p53 mutants were confirmed by DNA sequencing.

### RLIM Promoter Deletion and Mutation Constructs

By PCR amplification using primers 5′- ATAGGTACCTGGCCAGGCTGGTCTC -3′ and 5′-ATAGCTAGCGACAGGAAACGACTGC-3′, we generated a 600 bps fragment from −500 to +100 in the 5′-flanking untranslated region of the human *RLIM* gene. Then the fragment was digested with *Kpn* I and *Nhe* I, and ligated into the pGL3 Luciferase Reporter Vector (Promega) to generate the NP500-Luc construct. All the RLIM promoter 3′-5′ serial deletion constructs were generated by PCR amplification using NP500-Luc as the template with the forward primer, 5′-ATAGGTACCTGGCCAGGCTGGTCTC-3′ and the following reverse primers: 5′- ATAGCTAGCCCTCGAAAAGGCTCCG -3′ (NP500-DN100-Luc); 5′- ATAGCTAGCGGCAAGGCGGGCGGCG-3′ (NP500-DN150-Luc); 5′- ATAGCTAGCACCATGTGACAG-3′ (NP500-DN200-Luc) and 5′- ATAGCTAGC GCTGAGAATTGTGGG -3′ (NP500-DN250-Luc). Then all the PCR products were digested with *Kpn* I and *Nhe* I, and ligated into the pGL3 Luciferase Reporter Vector (Promega). RLIM promoter constructs with single or multiple Sp1 binding site mutations were generated using the QuikChange Site-Directed Mutagenesis Kit (Stratagene). Primers used to mutate Sp1 binding sites S1-S4 were as follows: M1-Forward, 5′- CAAAATGGCGGAGTAGTTTTGTTCCTGTCACATGGTG -3′; M1-Reverse, 5′- CACCATGTGACAGGAACAAAACTACTCCGCCATTTTG -3′; M2-Forward, 5′-CCTTTTC.

CAAACCGTTGCTTGTCTTGTCGCGTCGTCTG -3′; M2-Reverse**,** 5′- CAGACGACGCGACAAGACAAGCAACGGTTTGGAAAAGG -3′; M3-Forward, 5′- GTCTGCCGTCCTTCTTTTCCTCTTGCGGAGC -3′; M3-Reverse, 5′- GCTCCGCAAGAGGAAAAGAAGGACGGCAGAC -3′; M4-Forward, 5′-GCTAATAGGCTGTTAGTTTGTCTGTGGTGCCGGGAC -3′ and M4-Reverse,5′- GTCCCGGCACCACAGACAAACTAACAGCCTATTAGC-3′. All the wild type, serial deletions and mutated RLIM promoter luciferase reporter constructs were confirmed by DNA sequencing. A p53-Luc luciferase reporter plasmid containing 15 repeats of consensus p53 binding sites was used as the control (Stratagene).

### Transient Transfections and Luciferase Reporter Assays

For transient transfections, 5.0 × 10^4^ H1299 cells per well were plated into a 24-well plate and grown overnight. Transfections with Lipofectamine2000 (Invitrogen) were performed according to the manufacturer’s instructions. Briefly, increasing amounts of wt-pCMV-HA-p53 plasmid or pCMV-Myc-Sp1 plasmid were cotransfected with various RLIM promoter reporter constructs (NP-Luc series) together with pRL-SV40 plasmid as an internal control into H1299 cells. The total amount of plasmid was adjusted to equal with the empty vector plasmid. The Opti-MEM medium (Life Technologies, Inc.) was changed for RPMI 1640 supplemented with 10% fetal bovine serum 5 h after transfection. Cells were harvested 30 h after transfection, washed gently with cold phosphate-buffered saline and lysed in 120 µl reporter lysis buffer (Promega) for 15 min at room temperature. Cell lysates were centrifuged at 12,000 rpm for 1 min and the aliquots (10 µl) of cell extracts were analyzed for luciferase activity using the Dual Luciferase Assay System Kit (Promega) according to the manufacturer’s instructions. Values are the mean ±S.D. of relative luciferase units (RLU) from triplicate samples in three independent experiments normalized to pRL-SV40 activity.

### Western Blot Analysis and Antibodies

Cells were harvested 40 h after transfection, rinsed with phosphate-buffered saline and lysed in 1×SDS lysis buffer. Proteins were fractionated by electrophoresis on 10% SDS-polyacrylamide gels and transferred onto nitrocellulose membranes. After blocking with 5% nonfat milk in TBS-T Buffer for 1 h, the membranes were incubated with indicated antibodies for 2 h at room temperature, rinsed with TBS-T and incubated with secondary antibodies conjugated with horseradish peroxidase for 1 h at room temperature. Blots were developed by chemiluminescence (ECL, Santa Cruz) and subsequently exposed to X-ray films. Monoclonal anti-p53 antibody DO-1 was purchased from Santa Cruz Biotechnology. Monoclonal anti-HA antibody was purchased from Roche. Monoclonal anti-Myc, and anti-β-actin antibodies were purchased from Sigma. Polyclonal anti-RLIM antibody was made ourselves. The secondary HRP-conjugated goat anti-mouse IgG and goat anti-rabbit IgG antibodies were obtained from Calbiochem.

### Immunoprecipitation (IP) Studies

Cells were harvested at 40 h after transfection, washed with ice-cold PBS and resespended in lysis buffer (Cell Signaling) containing proteases inhibitor cocktail. After incubation for 30 min at 4°C with rotation, the cell lysates were centrifuged at 10,000 rpm for 20 min at 4°C and pre-cleared with 30 µl of protein A/G sepharose. The immunoprecipitation was performed by incubating the supernatants with 2 µg of anti-Myc antibody (Sigma) at 4°C overnight on a rotating platform. Then 40 µl of protein A/G sepharose were added and incubated at 4°C for 1 h. After washing 5 times with lysis buffer, the beads were lysed in equal volume of 2 × SDS lysis buffer and subjected to western blot analysis as described above.

### Treatment of Cells with DNA damaging Agent or siRNA and Quantitative PCR (qPCR)

For DNA damaging agent treatment experiments, U2-OS cells (p53+/+) were treated with 20 µM etoposide (Sigma) for the indicated period (0 h, 6 h and 12 h) to induce the expression of endogenous p53. For RNAi experiments, U2-OS cells were transfected with 20 µM p53 siRNA or NS (non-specific) siRNA as a control (GenePharma). Total RNA was isolated using Trizol (Invitrogen) according to the manufacturer’s instructions. cDNA synthesis was performed using the SuperScrip II reverse transcriptase Kit (Invitrogen) according to the manufacturer’s instructions. qPCR was conducted in triplicate in 10 µl volume using SYBR Green PCR Master Mix (Toyobo) with a Roche Lightcycler 480 II Real-Time PCR system. The following procedures were used: 6 min at 95°C for initial denaturing, followed by 40 cycles of 95°C 15 s, and 60°C 35 s. The following oligonucleotides were used to analyze the mRNA levels of RLIM, p53, p21^WAF1/CIP1^ and β-actin (internal control), respectively: RLIM-Forward, 5′- AGAGTTGCTGAGACGACTACAG-3′; RLIM-Reverse, 5′- TAGAACGTCTTGCAGATGGCTC -3′; p53-Forward, 5′- CATCCTCACCATCATCACACTG, p53-Reverse, 5′- TGGACTTCAGGTGGCTGGAGTG-3′, p21-Forward, 5′- ATGTCAGAACCGGCTGGGGATG -3′; p21-Reverse, 5′-TTAGGGCTTCCTCTTGGAGAAG -3′; β-actin-Forward, 5′- TACCACTGGCATCGTGATGGAC-3′ and β-actin-Reverse, 5′- GATCTCCTTCTGCATCCTGTCG -3′. The PCR products were subjected to electrophoresis on 1% agarose gel and visualized with ethidium bromide (EB) staining under UV light. All results were repeated in at least three independent experiments.

### Electrophoretic Mobility Shift Assays (EMSA)

EMSA analysis was performed using DIG Gel Shift Kit, 2nd Generation (Roche), or using LightShift Chemiluminescent EMSA Kit (Thermo scientific) according to the manufacturer’s instructions, respectively. When using DIG Gel Shift Kit, the RLIM promoter fragments containing each putative Sp1 binding site were prepared by PCR using NP500-Luc construct as a template. After purification, the PCR products were labeled by DIG as probes. The following primers were used in PCR: EMSA-0, 5′- GACAGGAAACGACTGCCCAC-3′; EMSA-50-A, 5′- GCGGTGCCGGGACGCGGCATCATCG-3′; EMSA-50-B, 5′- ATGATGCC.

GCGTCCCGGCACCGC -3′; EMSA-100-A, 5′-CCCGCGGAGCCTTTTCGAGC -3′; EMSA-100-B, 5′- CGATTCCTCGAAAAGGCT -3′; EMSA-150-A, 5′-ATCCAAACCGCCGCCCGCCTTG -3′; EMSA-150-B, 5′-CGGCAAGGCGGGCGGCGGTTTGG -3′; EMSA-200-A, 5′- ATGGCGGAGGAGGGTGGGGCC -3′; EMSA-200-B, 5′- TGACAGGCCCCACCCTCCTC -3′; EMSA-250-A, 5′- GGTTCCCACAATTCTCAGAACGC -3′; EMSA-250-B, 5′- GCGTTCTGAGAATTGTGGGAACC -3′; EMSA-300-A, 5′- CAAGCAGTCAGACCGTGACG-3′ and EMSA-300-B, 5′- CGTCACGGTCTGACTGCTTG -3′. For DNA binding reactions, DIG labeled probes were incubated in the presence or absence of 150 ng recombinant human Sp1 protein (rhSp1, Promega) in standard 20 µl binding mixtures (DIG Gel Shift Kit, Roche). After incubation at room temperature for 25 min, the samples were analyzed by electrophoresis on a nondenaturing 6% polyacrylamide gel and imaged by chemiluminescent detection. The competition binding assay was performed with 50-fold excess of unlabeled cold competitor oligonucleotides as indicated. To detect the effect of p53 on binding of Sp1 protein to the RLIM promoter, EMSA was performed by incubating the DIG-labeled probe spanning the −100/+50 region of the RLIM promoter containing three putative Sp1 binding sites (S2, S3 and S4) with either 150 ng rhSp1 protein alone or together with increasing amounts (50 ng, 100 ng) of wild type p53 protein. The electrophoresis and chemiluminescent detection was performed as described above. When using the LightShift Chemiluminescent EMSA Kit (Thermo Scientific), the Biotin-labeled probes were purchased from Sangon biotech. For Sp1 mutated probes, the Sp1 binding elements were replaced by TTTTTA. The procedures were conducted according to the manufacturer’s instruction, which were similar to the DIG Gel Shift Kit.

### Chromatin Immunoprecipitation Assays (ChIP)

ChIP assays were performed using the ChIP Assay Kit (Upstate) according to the manufacturer’s instructions. A total of 1 × 10^6^ cells fixed with 1% formaldehyde for 10 min at 37 ^o^C. Cross linking was stopped by adding glycine to a final concentration of 125 mM. The cells were washed twice with ice cold PBS containing proteases inhibitor cocktail, scraped and centrifuged for 4 min at 2000 rpm at 4 ^o^C. Cell pellets were resuspended in SDS Lysis Buffer, incubated for 10 min on ice and sonicated at 4°C to shear DNA to an average fragment size of 500 basepairs. After centrifugation for 10 min at 13,000 rpm at 4°C, the supernant was diluted 10 fold in ChIP Dilution Buffer containing protease inhibitor cocktail and pre-cleared. Then the anti-Sp1 antibody (Santa cruz), anti-Myc antibody (Sigma), anti-HA antibody (Roche) or control IgG antibody was added as indicated and incubated overnight at 4°C with rotation. The immune complexes formed were collected by Protein A Agarose/Salmon Sperm DNA and incubated for 2 h at 4°C with rotation. After gentle centrifugation, the agarose beads were sequentially washed on a rotating platform with the following buffers, Low Salt Wash Buffer, High Salt Wash Buffer, LiCl Wash Buffer, and finally TE Buffer for twice. The Protein/DNA complex were eluted with the elution buffer (1% SDS, 0.1 M NaHCO_3_). To reverse the crosslinks, 20 µl of 5 M NaCl were added and heated at 65°C for 4 hours. After addition of 2 µl of 10 mg/ml Proteinase K, the samples were incubate for 1 h at 45°C. DNA recovered from immuno-complexes and input material were purified by phenol/chloroform extraction and ethanol precipitation, and subsequently subjected to PCR analysis using primers to the −200/+50 region of RLIM promoter (Forward, 5′- CAAGCAGTCAGACCGTGACG -3′; Reverse, 5′-ATGATGCCGCGTCCCGGCACCGC-3′, primers to RLIM upstream irrelevant regions (Forward, 5′- TGGCAATTGCAGGCTACTGA; Reverse, 5′- GGGAGAATTACCCCCTCACC), or primers to the p21 promoter as positive control (Forward, 5′-CTGGACTGGGCACTCTTGTC-3′; Reverse, 5′- CTCCTACCATCCCCTTCCTC-3′). The PCR products were separated by agarose gel electrophoresis and visualized by EB staining.

## Results

### Repression of the RLIM Promoter Activity by Wild Type p53

**Figure 1:**
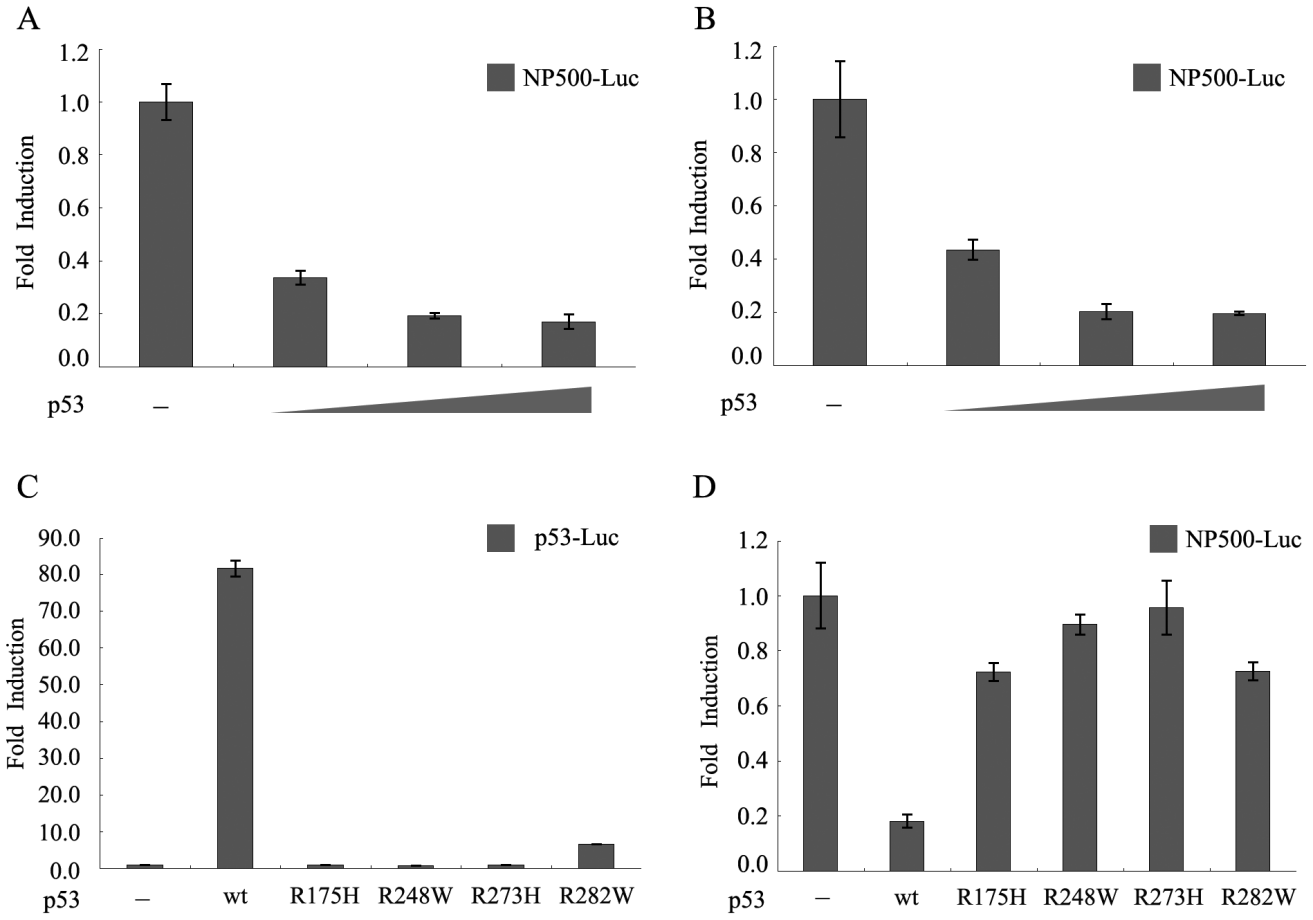
Dose-dependent repression of the RLIM promoter by p53. (**A**) H1299 cells or (**B**) Hep3B cells were cotransfected with 100 ng of RLIM promoter-luciferase reporter construct NP500-Luc, 30 ng pRL-SV40 and increasing amounts (0, 1, 2.5 and 10 ng) of pCMV-HA-p53 plasmids. The total amount of plasmid in each transfection was adjusted to be the same using the empty vector. Cells were harvested 30 h after transfection and lysed for measuring luciferase activity. Data represent the mean of three independent experiments normalized to pRL-SV40 activity and are presented as fold of induction; bars, ± S.D. (**C**) Various p53 expression constructs (wild type and mutants with mutations in codons 175, 248, 273 and 282) or pCMV-vector plasmid were cotransfected with 100 ng of p53-Luc luciferase reporter gene (Stratagene). Cells were harvested 30 h after transfection and lysed for luciferase assays. (**D**) Effects of p53 and various mutants on RLIM promoter activity. The indicated p53 expression plasmids or pCMV-vector plasmid were transfected with 100 ng of RLIM promoter-luciferase plasmid NP500-Luc. Cells were harvested 30 h after transfection and lysed for luciferase assays.

To investigate the transcriptional regulation of the RLIM promoter activity by p53, we performed luciferase reporter assays in a p53 null cell line H1299. The luciferase reporter construct, NP500-Luc, contained the −500/+100 region of the RLIM promoter in front of the luciferase reporter gene. Cotransfection of NP500-Luc and increasing amounts of wild type p53 expression plasmid resulted in repression of RLIM promoter activity in a dose-dependent manner with the maximal repression at 84% (Figure. 1A). Further increase in the p53 expression plasmid over 10 ng did not cause further inhibition. Similar repression of the RLIM reporter gene by wt-p53 was also observed in Hep3B cell lines (p53 null), indicating that the inhibitory effect on RLIM promoter activity by p53 is not restricted to a specific cell type (Figure. 1B).

We generated a series of p53 mutant constructs (R175H, R248W, R273H and R282W) and determined their effects on the RLIM luciferase reporter gene in comparison with wild type p53. All of the p53 mutants have lost transactivation capabilities as indicated in Figure. 1C by luciferase reporter assays using a p53-Luc luciferase reporter plasmid (Stratagene). Interestingly, only wild type p53 repressed the RLIM reporter, whereas none of the p53 mutants had any effect on the same reporter under identical conditions (Figure. 1D). These results suggested that the integrity of the DNA binding domain of p53 was essential for the repression of RLIM promoter activity.

### RLIM is Repressed at Both mRNA and Protein Levels by p53

**Figure 2:**
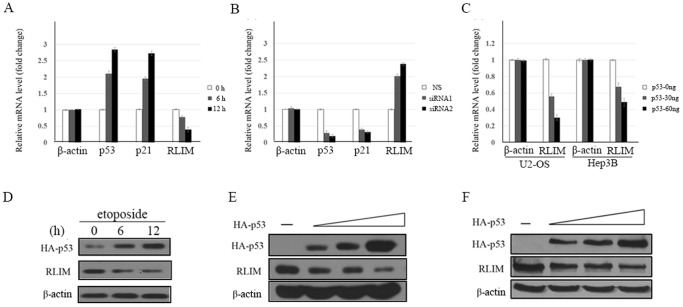
p53 represses RLIM at both mRNA and protein levels. (**A**) U2-OS cells were treated with 20 µM etoposide for the indicated period (0, 6 and 12 h) to induce the expression of endogenous p53. Cells were harvested for total RNA isolation followed by qPCR using primers specific for RLIM, p21, p53 and β-actin mRNA, respectively. (**B**) U2-OS cells were transfected with 20 µM p53 siRNA or 20 µM NS (non-specific) siRNA as control. RNA isolation and qPCR were performed as described above. (**C**) U2-OS or Hep3B cells were transfected with increasing amounts of p53 and harvested 24 h after transfection. Cells were harvested for total RNA isolation followed by qPCR using primers specific for RLIM and β-actin respectively. (**D**) U2-OS cells were treated with 20 µM etoposide for the indicated period (0, 6 and 12 h) to induce the expression of endogenous p53. Cells lysates were immunoblotted with antibodies to p53, RLIM and β-actin. (**E**) U2-OS or (**F**) Hep3B cells were transfected with different amount of plasmids encoding p53. Cells were harvested 40 h after transfection and lysates were immunoblotted with antibodies to p53, RLIM and β-actin.

The qPCR and Western blot were performed using the human osteogenic sarcoma cell line U2-OS (p53^+/+^) to examine whether p53 regulates RLIM mRNA and protein level. When treated with etoposide, a DNA damaging agent, to induce the expression of endogenous p53, RLIM was reduced in a time-dependent manner (Figure. 2A, Figure. 2D). As control, the mRNA level of known p53 target gene p21 was upregulated after etoposide treatment. In a complementary experiment, when endogenous p53 was knocked down, the RLIM mRNA level increased (Figure. 2B). Thus, p53 is a negative regulator of RLIM.

We then transfected Hep3B cells (p53−/−) and U2-OS cells (p53+/+) with increasing amounts of p53 to examine the effect of enforced expression of p53 on endogenous RLIM level. As the expression of p53 increased, the level of endogenous RLIM mRNA and protein was reduced in a dose-dependent manner (Figure. 2C, Figure. 2E, Figure. 2F). These results indicated that p53-dependent repression of RLIM occurs at both mRNA and protein levels.

### Four Sp1 Elements Were Identified on the RLIM Promoter

**Figure 3:**
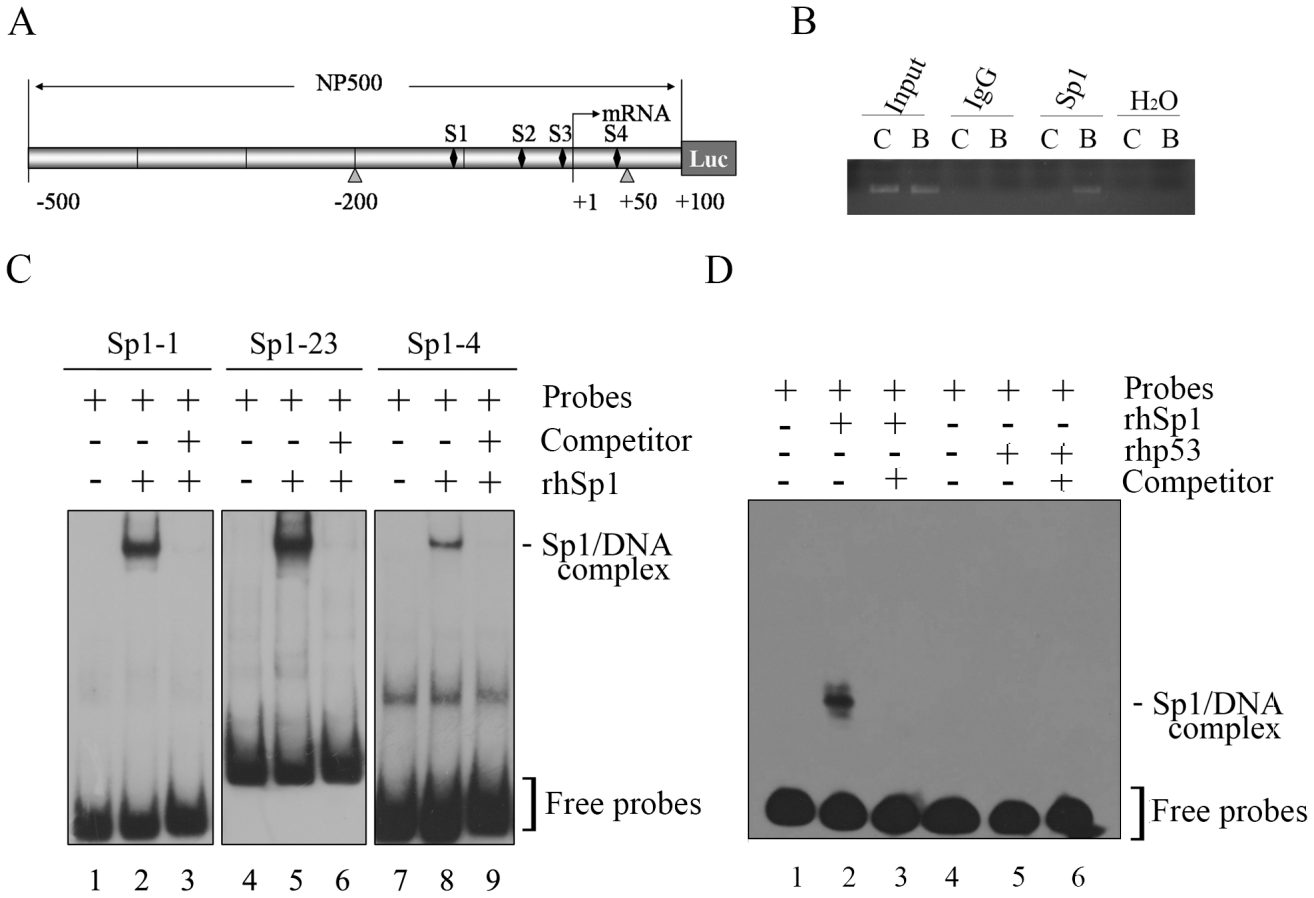
Sp1 Binds to the RLIM Promoter both *in Vitro* and *in Vivo*. (**A**) Schematic representation of the RLIM promoter region in NP500-Luc. The four putative Sp1 binding sites S1 to S4 (denoted as the solid bar⧫) are shown. The arrow indicates the transcription start site. The triangles mark the flanking sites of the PCR fragment amplified in the ChIP assays (–200 bp to +50 bp). (**B**) ChIP analysis for the binding of endogenous Sp1 to the RLIM promoter. Chromatin immunoprecipitations (ChIP) were performed with antibodies against either IgG as control or Sp1. DNA recovered from immunocomplexes were subjected to PCR using primers flanking Sp1 binding region, or primers corresponding to irrelevant upstream chromatin region. PCR was performed without chromatin as negative control (B, primers corresponding to binding region. C, primers corresponding to irrelevant upstream chromatin region). (**C**) EMSA analysis of the binding of Sp1 to the RLIM promoter *in vitro*. EMSA was performed using DIG-labeled PCR fragments of the RLIM promoter containing each putative Sp1 binding site as indicated. DIG labeled probes were incubated in binding reactions in the presence or absence of 150 ng rhSp1 protein (Promega). Lanes 1, 4 and 7: probes only. Lanes 2, 5 and 8: rhSp1+ probes. Lanes 3, 6 and 9: rhSp1+ probes +50-fold excess cold unlabeled competitor DNA. Bands for the Sp1/DNA complexes and free probes are indicated. (**D**) EMSA analysis of the binding of Sp1 or p53 to the RLIM promoter region using the LightShift Chemiluminescent EMSA Kit. Lanes 1, 4 : Biotin-labeled probes only. Lane 2: rhSp1+ Biotin-labeled probes. Lane 3: rhSp1+ Biotin-labeled probes +50-fold excess cold unlabeled competitor DNA. Lane 5: rhp53+ biotin-labeled probes. Lane 6: rhp53+ biotin-labeled probes +50-fold excess cold unlabeled competitor DNA.

To gain insight into the mechanism of the p53-mediated repression of RLIM, we analyzed the −500/+100 region of the RLIM promoter for potential transcription factor elements that may be involved in the functional regulation. Interestingly, inspection of the RLIM promoter showed no consensus p53 binding sites, ruling out the possibility that p53 repressed RLIM transcription by directly occupying its promoter region. p53 has been shown to repress transcription by multiple mechanisms, and the p53 consensus binding site is dispensable for the repression mediated by p53 [5]. In this regard, p53 can exert the inhibitory effect by interfering with the functions of transcriptional activators, such as Sp1 or ETS1 [16]–[18]. By bioinformatics analysis, four putative Sp1 elements were identified in the RLIM promoter spanning nucleotides –109/−98, –52/−42, –23/−14 and +36/+46, designated S1–S4, raising the possibility that Sp1 might be involved in the functional regulation of the RLIM promoter by p53 (Figure. 3A).

### Sp1 Transcription Factor Binds to the RLIM Promoter Both in Vitro and in Vivo

Sp1 is a sequence-specific transcription factor. To examine whether Sp1 directly binds to the RLIM promoter, electrophoretic mobility shift assays (EMSA) were performed using recombinant human Sp1 protein (rhSp1, Promega) and DIG-labeled probes of RLIM promoter fragments containing each putative Sp1 binding site (as described under experimental procedures). A clear Sp1/DNA complex band was observed when Sp1 protein was added (Figure. 3C, compare lanes 1 and 2, lanes 4 and 5, lanes 7 and 8, respectively), indicating that Sp1 protein directly binds to all four putative Sp1 elements. However, the binding affinity of Sp1 for the four Sp1 sites differed significantly. Sp1 protein had the highest affinity for S2 and S3 sites, moderate affinity for S1 site, and the lowest affinity for S4 site. Cold unlabeled oligonucleotides were used as competitors to confirm the specificity of the binding of rhSp1 to the RLIM promoter. When 50-fold cold unlabeled oligonucleotides were added, they significantly reduced the RLIM promoter DNA/Sp1 complex bands (Figure. 3C, lanes 3, 6 and 9). We also performed EMSA using recombinant human p53 protein (rhp53) and Biotin-labeled probes of RLIM promoter fragment, and the result showed that unlike Sp1, p53 did not bind to the RLIM promoter region directly (Figure. 3D, compare lanes 2 and 5).

To verify the binding of Sp1 to the RLIM promoter in vivo, chromatin immunoprecipitation (ChIP) analysis was performed. Hela cells were fixed with 1% formaldehyde to crosslink protein/DNA complexes. After sonication to shear DNA and dilution of supernant in ChIP Dilution Buffer, immunoprecipitation was carried out using anti-Sp1 antibody (Santa Cruz) or IgG antibody as control. DNA recovered from immuno-complexes and input materials were subjected to PCR using primers covering the –200/+50 region that contains the four Sp1 elements or primers corresponding to irrelevant upstream region. As shown in Figure. 3B, Sp1 specifically bound to the RLIM promoter in vivo. Taken together, these results indicated that Sp1 transcription factor directly binds to the RLIM promoter both *in vitro* and *in vivo*.

### p53 Abrogates Sp1-mediated Activation of the RLIM Promoter

**Figure 4:**
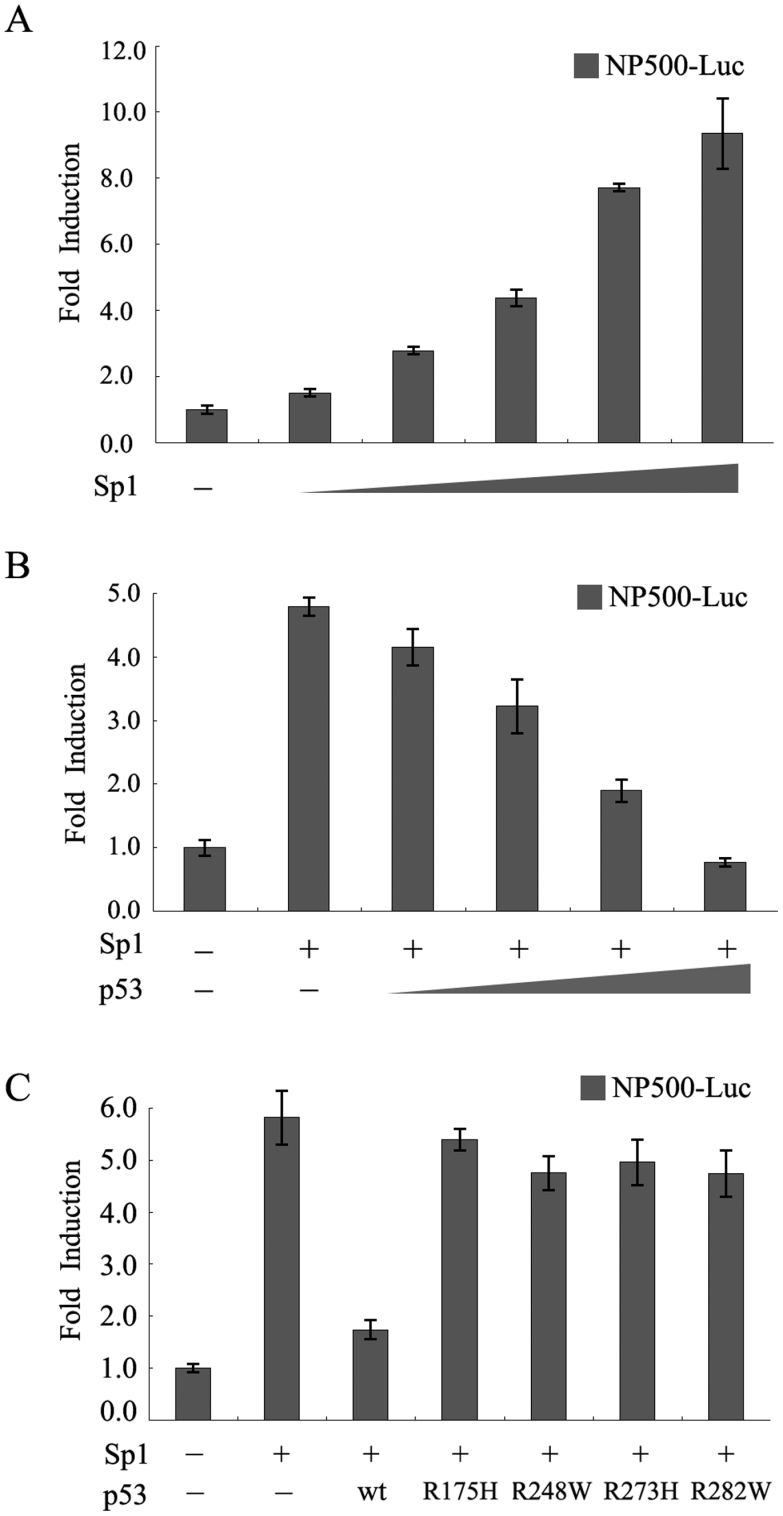
p53 inhibits Sp1-mediated activation of the RLIM promoter. (**A**) Sp1 activated the RLIM promoter activity. H1299 cells were cotransfected with 100 ng of NP500-Luc with either 160 ng pCMV empty vector as control or increasing amounts (0, 10, 20, 40, 80 and 160 ng) of pCMV-Myc-Sp1 plasmid. The total amount of plasmid in each transfection was adjusted to be the same using the empty vector. Cells were harvested 30 h after transfection and lysed for measuring luciferase activity. (**B**) p53 inhibits Sp1-stimulated activity of the RLIM luciferase reporter. H1299 cells were cotransfected with 100 ng of NP500-Luc and increasing amounts (0, 1, 2.5, 5 and 10 ng) of pCMV-HA-p53 plasmid in the presence of a fixed amount (160 ng) of pCMV-Myc-Sp1 plasmid. H1299 cells were transfected with 100 ng NP500-Luc alone as control. Cell lysates were used for luciferase assays as described above. (**C**) p53 mutants can not inhibit Sp1-stimulated activity of the RLIM luciferase reporter. The indicated p53 wild type or mutant constructs were cotransfected with 100 ng of RLIM promoter reporter construct NP500-Luc and 160 ng pCMV-Myc-Sp1 plasmid. Cells were harvested 30 h after transfection and the cell lysates were prepared and used for luciferase assays as described above.

To determine whether Sp1 can activate the RLIM promoter activity, we co-transfected H1299 cells with NP500-Luc luciferase reporter gene and increasing amounts of pCMV-Myc-Sp1 expression construct. As expected, the RLIM promoter activity was significantly activated by Sp1 in a dose-dependent manner compared with the control vector (Figure. 4A). The activation of RLIM promoter is up to 10-fold when 100 ng pCMV-Myc-Sp1 expression construct was used.

To further investigate the effect of p53 on Sp1-mediated activation of RLIM promoter activity, H1299 cells (p53 null) were transiently transfected with NP500-Luc alone or with fixed amount of pCMV-Myc-Sp1 expression construct and increasing amounts of wild type p53 expression plasmid. Wild type p53 was able to inhibit Sp1-mediated stimulation of RLIM promoter activity in a dose-dependent manner (Figure. 4B). In contrast to wild type p53, various p53 mutants failed to reduce Sp1-mediated stimulation of RLIM promoter activity (Figure. 4C), which was in agreement with the observation that p53 mutants were incapable of repressing the RLIM promoter activity (Figure. 1D). Taken together, these results suggested that p53 might repress RLIM promoter by interfering with the activity of Sp1.

### Sp1 Elements are Required for Activation of the RLIM Promoter

**Figure 5:**
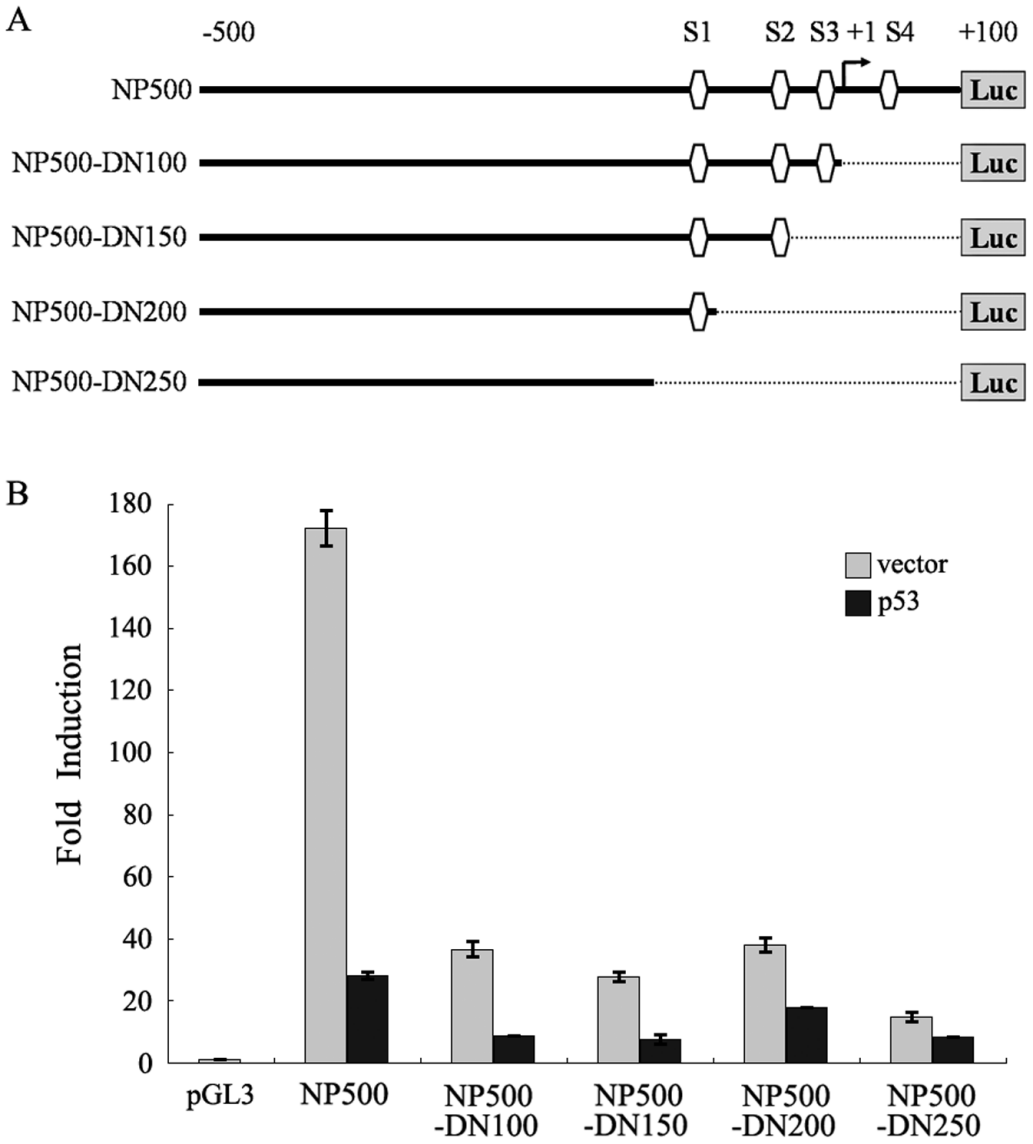
Identification of the essential RLIM promoter regions required for p53 mediated transrepression. (**A**) Schematic representation of the RLIM promoter luciferase reporter NP500-Luc (−500/+100) and various 3′ serial deletions luciferase reporter constructs of the RLIM promoter (NP500-DN 100 to NP500-DN250). The four putative Sp1 binding sites S1 to S4 are highlighted (denoted as the ). The broken line (**…**) indicates the deleted region of individual construct. The arrow indicates the transcription start site. (**B**) Various RLIM promoter luciferase constructs described in Figure. 5A or control pGL3 vector were transfected into cells for luciferase reporter assays. For p53, 10 ng of pCMV-HA-p53 plasmid was used. For vector, 10 ng of pCMV-vector plasmid was used. Aliquots of cell extracts were assayed for luciferase activity as described above.

To determine whether Sp1 binding sites are required for activation of the RLIM promoter, we generated a series of 3′–5′ deletion constructs of the RLIM promoter missing a subset or all Sp1 elements. The NP500-DN100, NP500-DN150, NP500-DN200 and NP500-DN250 luciferase reporter constructs lacked S4, S3S4, S2S3S4 and S1–S4 binding sites, respectively (Figure. 5A). The basal luciferase activities of the deletion mutants were significantly decreased compared with that of NP500-Luc, suggesting that Sp1 plays an essential role in the activation of the RLIM promoter. In addition, as more Sp1 elements were deleted, the p53-mediated repression of RLIM promoter activity were gradually abrogated (Figure. 5B). Thus, the transcription factor Sp1 is likely the key target of p53 for its repression of RLIM transcription.

**Figure 6:**
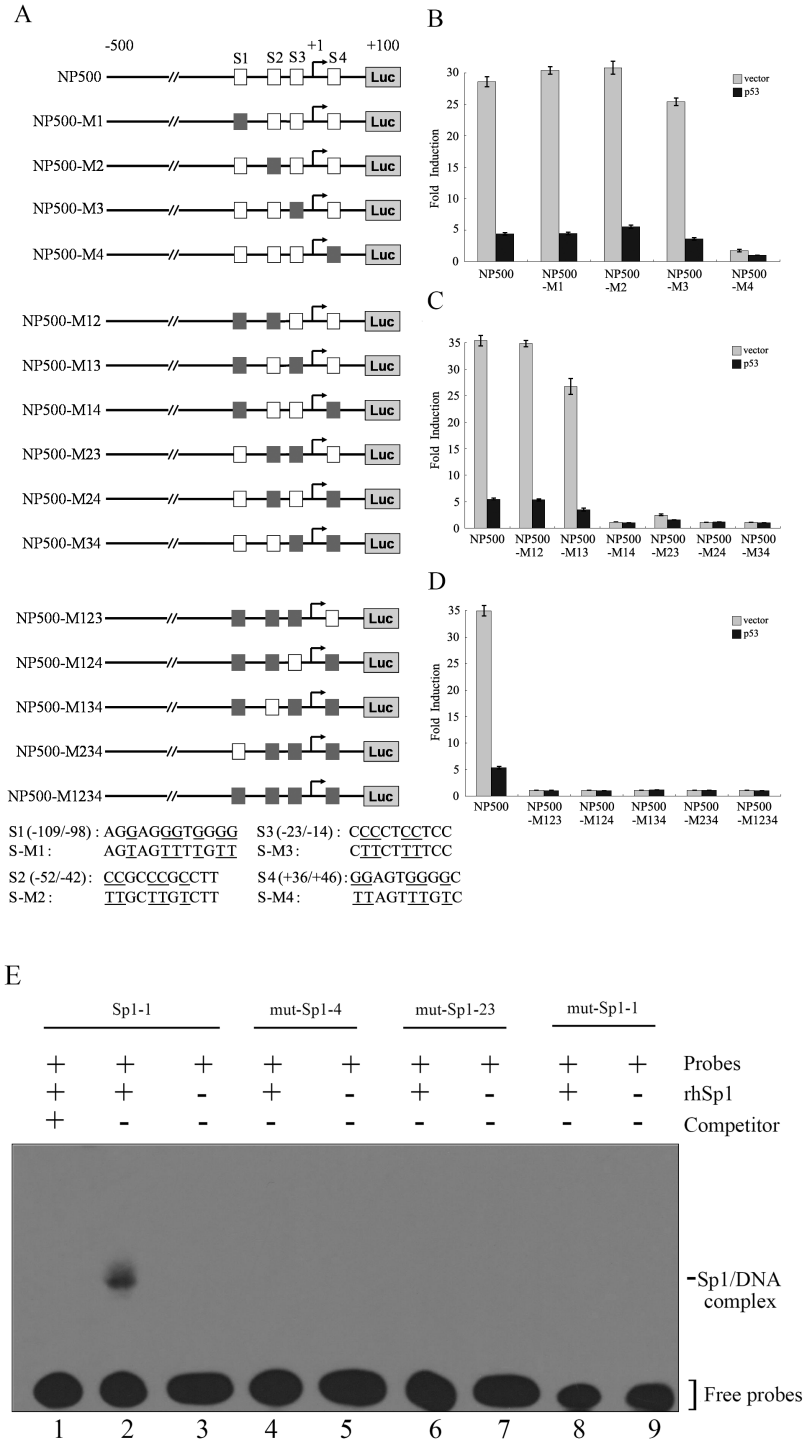
Sp1-binding sites mutations abrogate the repression of RLIM promoter by p53. (**A**) Schematic representation of the RLIM promoter luciferase reporter construct NP500-Luc and various mutated constructs with the Sp1 binding sites mutations (NP500-M1 to NP500-M1234). The four Sp1 binding sites S1 to S4 (wild type denoted as □ and mutations denoted a ▪) are shown. The sequences of the Sp1 binding sites are shown in the lower panel with the mutated sites underlined. (**B–D**) Cotransfection was performed as described in Figure. 5B using NP500-Luc and various mutated constructs with indicated Sp1-binding sites mutations, respectively. Luciferase assays were performed as described above. (**E**) EMSA analysis of the binding of Sp1 to RLIM promoter with different Sp1 binding site mutations (M1–M4). EMSA was performed as described in Figure. 3D. Lane 1: rhSp1+Biotin-labeled probes containing wild type Sp1-1 binding site +50-fold excess cold unlabeled competitor DNA. Lane 2: rhSp1+Biotin-labeled probes containing wild type Sp1-1 binding site. Lane 3: biotin-labeled probes containing wild type Sp1-1 binding site only. Lanes 4, 6, 8: rhSp1+Biotin-labeled probes containing mutant Sp1 binding site. Lanes 5, 7, 9: Biotin-labeled probes containing mutant Sp1 binding site only.

To further analyze the role of the Sp1 elements in the regulation of RLIM promoter activity, we generated various Sp1 element mutants and determined the luciferase reporter activities in the absence and presence of p53 (Figure. 6A). Of the four putative Sp1 sites, only mutation of S4 significantly decreased the basal activity of RLIM promoter to nearly background (Figure. 6B). When two Sp1 elements were mutated in combination, the S2–S3 combination led to dramatically diminished reporter activity in addition to other combinations involving S4 (Figure. 6C). All mutants in which three or all four Sp1 elements were mutated showed little activity (Figure. 6D). To further examine the binding of Sp1 transcription factor to the RLIM promoter, EMSA were performed using rhSp1 protein and Biotin-labeled probes of RLIM promoter fragment containing wild type Sp1-1 binding site or each mutated Sp1 binding site. The results showed that the mutation of Sp1 elements prevented the binding of Sp1 protein to RLIM promoter (Figure. 6E). Taken together, these results further demonstrated that the Sp1 binding elements, are essential for RLIM transcription.

### p53 Interacts with Sp1 and Inhibits Its Binding to the RLIM Promoter

**Figure 7:**
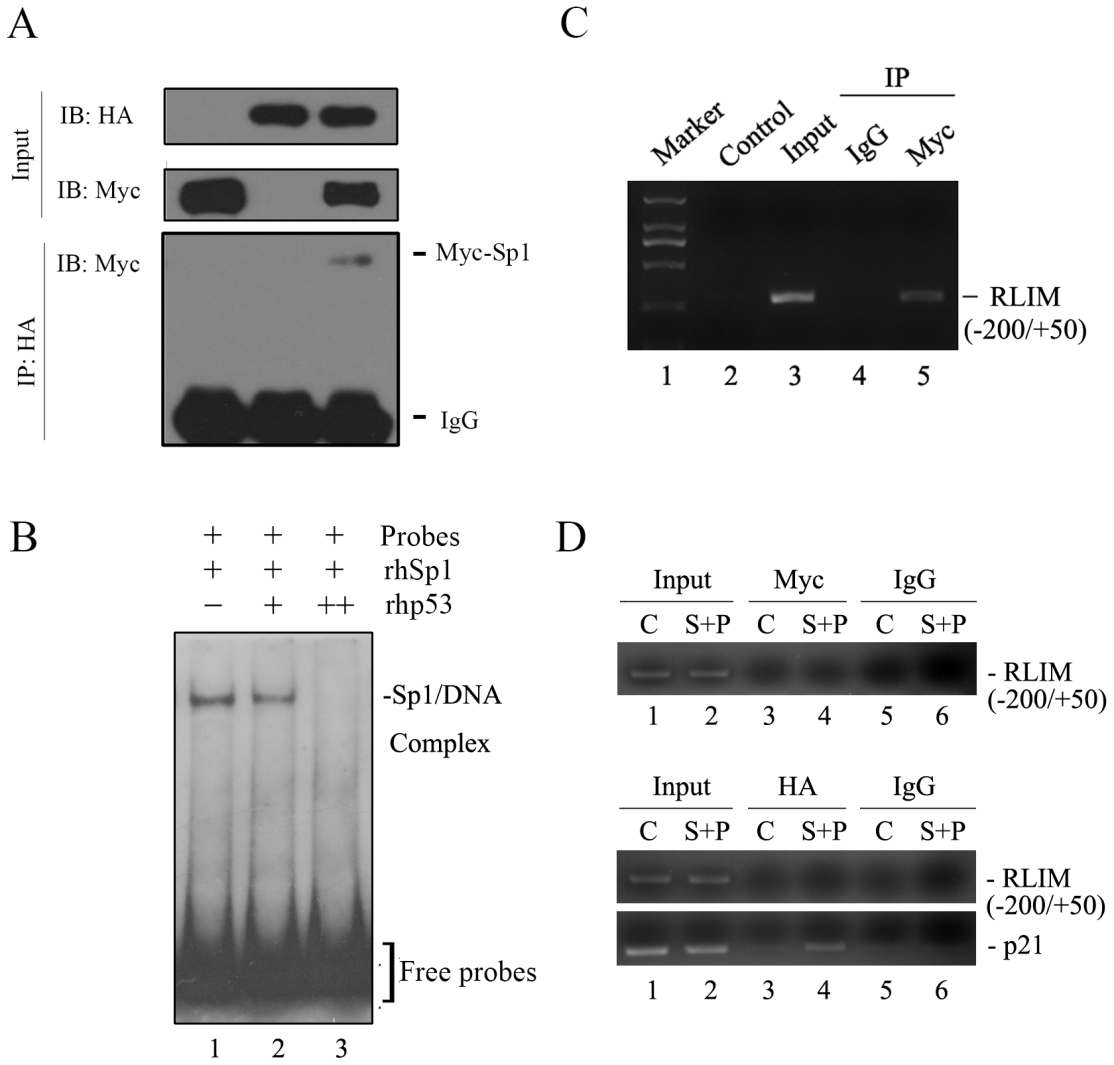
p53 interacts with Sp1 and inhibits its binding to the RLIM promoter. (**A**) H1299 cells were transfected with pCMMV-HA-p53, pCMV-Myc-Sp1 alone or both as indicated. Whole-cell extracts were immunoprecipitated with anti-HA antibody and subjected to Western blot analysis with anti-Myc antibody. As control, 10% of the cell lysates were used as input. (**B**) Effect of p53 on Sp1 binding to the RLIM promoter. Purified recombinant Sp1 and p53 proteins were used for EMSA. The DIG-labeled probe spanning the –100/+50 region of the RLIM promoter containing three putative Sp1 binding sites (S2, S3 and S4) was incubated in binding reactions with either rhSp1 protein alone (Lane1) or together with increasing amounts of p53 protein (Lanes 2 and Lane 3). EMSA was performed as described above. Bands for the Sp1-DNA complex and free probes are indicated. (**C**) ChIP analysis for the binding of Sp1 to the RLIM promoter. Chromatin immunoprecipitations (ChIP) were performed with antibodies against either IgG as control or Myc. DNA recovered from immunocomplexes were subjected to PCR using primers flanking Sp1 binding region. (**D**) p53 prevents the binding of Sp1 to the RLIM promoter *in vivo*. The H1299 cells transfected with either control vector plasmid (C, lanes 1, 3 and 5) or pCMV-Myc-Sp1 together with pCMV-HA-p53 plasmids (S+P, lanes 2, 4 and 6) were subjected to ChIP assays using anti-Myc, anti-HA or IgG antibodies as indicated. DNA purified from input chromatin (Input) or immunocomplexes were subjected to PCR analysis using primers flanking the –200/+50 region of the RLIM promoter or p21 promoter as a positive control.

It has been reported previously that p53 directly interacts with Sp1 [17]–[20]. We verified the association between p53 and Sp1 *in vivo* by IP studies (Figure. 7A). Given that p53 repressed Sp1 transcriptional activation activity without direct binding to the Sp1 binding sites on RLIM promoter, we hypothesized that p53 might exert its inhibitory effect on Sp1 by interaction with Sp1 to either inhibit Sp1 binding to the RLIM promoter (exclusively sequestration) or blocks Sp1 transactivation capabilities through the formation of ternary complex at RLIM promoter. To distinguish between these possibilities, ChIP assays were performed in H1299 cells transfected with either control vector (C, lanes 1, 3 and 5) or pCMV-Myc-Sp1 together with pCMV-HA-p53 plasmids (S+P, lanes 2, 4 and 6) using anti-Myc, anti-HA or IgG antibodies as indicated. DNA purified from input chromatin or immunocomplexes were subjected to PCR analysis using primers flanking the four Sp1 binding sites on RLIM promoter (–200/+50) or p21 promoter as a positive control. As shown in Figure. 7D, in contrast to the apparent binding to RLIM promoter observed when Myc-Sp1 was transfected alone (Figure. 7C), neither Myc-Sp1 (Figure. 7D, upper panel, lane 4) nor HA-53 (Figure. 7D, lower panel, lane 4) was able to bind to the RLIM promoter when they were cotransfected into cells. As positive control, the DNA-binding activity of p53 to p21 promoter was normal (Figure. 7D, bottom panel). These results suggest that p53 could not directly bind to RLIM promoter or form the ternary complex with Sp1 at RLIM promoter. Instead, it appears to dissociate Sp1 from the RLIM promoter.

To further verify whether p53 directly blocked the binding of Sp1 to RLIM promoter, we performed competitive EMSA using DIG-labeled RLIM promoter fragment containing three major Sp1 elements S2, S3, and S4 and human recombinant Sp1 protein in the presence or absence of increasing amounts of recombinant p53 protein. As shown in Figure. 7B, p53 caused an inhibition of binding of Sp1 to the RLIM promoter in a dose-dependent manner. Once again, no p53/RLIM promoter DNA complex was detected in this experiment, supporting the notion that the p53-mediated repression of RLIM promoter activity does not require the direct binding of p53 to RLIM promoter, and is consistent with the results of luciferase reporter assays. Taken together, these results demonstrated that p53 repressed RLIM by blocking of Sp1 binding to the RLIM promoter and consequent interference with the Sp1-mediated transcriptional activation.

## Discussion

p53 is known to regulate a myriad of biological processes by modulating the expression of different target genes. In this study, we identified RLIM as a novel target of p53 whose expression is downregulated by p53. Further dissection of the regulatory mechanism revealed that the repression of RLIM transcription by p53 is mediated through inhibition of the binding of Sp1 to the RLIM promoter. These findings shed new light on the regulation of RLIM by p53 and suggested a new pathway by which physiological and pathological activators of p53 may affect development.

As a DNA-binding transcription factor, p53 is usually known to activate, rather than repress, the transcription of its target genes. Somewhat surprisingly, however, a genome-wide expression analysis using DNA microarrays revealed that more than 80% of the p53 target genes are repressed rather than activated [23]. To activate transcription, p53 binds to consensus responsive elements on the promoters of its target genes. In contrast, the promoters of p53-repressed genes usually lack p53 consensus binding sites [5]. One way p53 exerts its repressive effects is through direct interaction with transcriptional activators required for the transcription of its target genes. For example, it has been shown that p53 inhibits transcriptional activation of VEGF [16] and PKCα [17] by direct interaction with Sp1. Similar to VEGF and PKCα, no p53-binding sites were found throughout the RLIM promoter. Given that Sp1 has been shown to be involved in the transcriptional activation o RLIM, we determined whether Sp1 plays an indispensable role in RLIM transcription and if so, whether p53 downregulates RLIM through Sp1. Although RLIM is known to be regulated by several transcription factors in addition to Sp1, we found that Sp1 is required for the transcriptional activation of RLIM through analysis of site-directed mutations of the four Sp1 binding elements on RLIM promoter. We further demonstrated that p53 is capable of blocking the binding of Sp1 to RLIM promoter both *in vitro* and in vivo in cells using EMSA and ChIP assays, respectively. The simplest model consistent with those results is that p53 exerts its repressive effect on RLIM by binding to, and blocking the association of Sp1 to the RLIM promoter.

p53 has been shown to inhibit the activity of transcription factors in multiple ways. Li et al. reported that the addition of p53 protein abrogated the binding of Sp1 protein to the POLD1 promoter in an EMSA assay [24]. Unlike RLIM, however, a p53 consensus binding site was identified in the POLD1 promoter that overlapped the Sp1 binding site. And inhibition of Sp1 by p53 occurs at the level of competitive binding to the POLD1 promoter [24]. In contrast to the POLD1 promoter, we did not find any consensus p53 binding site on the RLIM promoter and could not detect p53/DNA complex, suggesting that p53 does not directly bind to the RLIM promoter to exert inhibitory effect (Figure. 7C). In this case, binding of Sp1 to p53 is mutually exclusive to its binding to RLIM promoter. Similar results on the regulation of the Sp family of proteins by p53 have been reported by others [19], [21], [22].

In addition to Sp1, p53 has been shown to directly interact with ETS1 and repress ETS1-mediated activation of target genes, such as IKKα [20] and TXSA [25]. In both cases, p53 exerts its inhibitory effect without direct binding to the target gene promoters. We have also found that p53 is capable of repressing ETS1-mediated activation on RLIM promoter (data not shown). It will be interesting to determine whether ETS1 also contribute to the inhibition of RLIM promoter activation by p53.

In addition to repressing RLIM expression at the transcription level, p53 also downregulates RLIM at protein level. RLIM has been previously reported to be targeted for degradation by the E3 ubiquitin ligase Siah-1, one of the human homologues of the *Drosophila* seven in absentia (Sina) protein [26], [27]. Interestingly, Siah-1 and Siah-1b (mouse homologue) were identified as p53-inducible genes that were upregulated during apoptosis and for tumor suppression [28]–[31]. Thus, p53 may repress the RLIM protein level by induction of its E3 ubiquitin ligase Siah-1.

**Figure 8:**
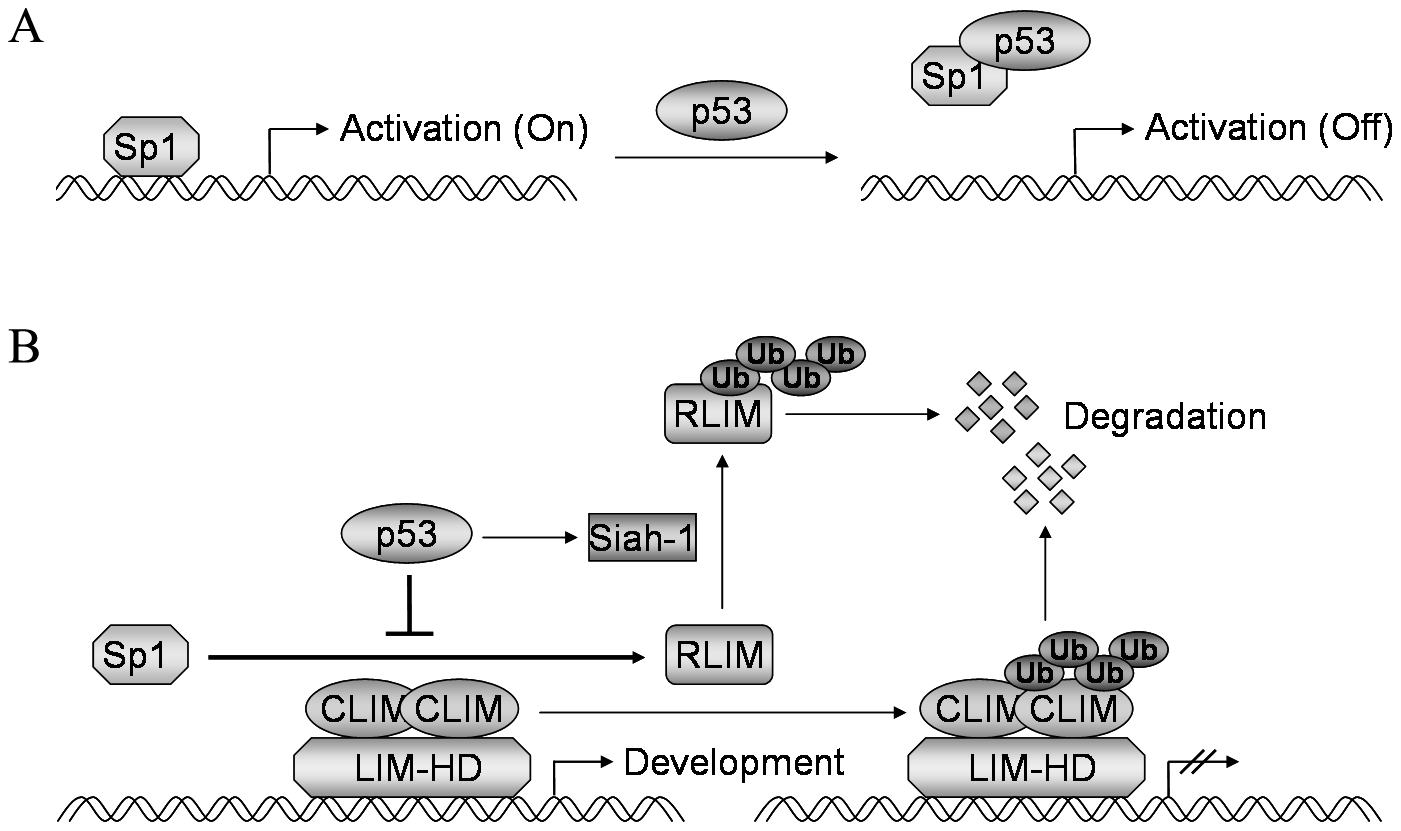
Mechanism of inhibition of RLIM by p53. (**A**) p53 directly interacts with and sequesters Sp1 from the RLIM promoter, thereby inhibiting the transcriptional activation of RLIM by Sp1. (**B**) Dual mechanism of inhibition of RLIM by p53. p53 inhibits the transcriptional activation of RLIM by Sp1. p53 also activates the expression of RLIM E3 ubiquitin ligase Siah-1, leading to the degradation of RLIM protein.

In summary, we identified RLIM as a novel target gene of p53, and demonstrated that p53 inhibits the transcription of RLIM through direct binding to Sp1 and its sequestration from the RLIM promoter (Figure. 8A). Concomitantly, p53 activates the transcription of the RLIM E3 ubiquitin ligase Siah-1, leading to the ubiquitination and degradation of existing RLIM proteins (Figure. 8B). Together, this dual mechanism of transcriptional repression and protein degradation ensures timely and complete elimination of RLIM by p53, which may be critical during development.
